# Human wild-type superoxide dismutase 1 gene delivery to rat bone marrow stromal cells: its importance and potential future trends

**DOI:** 10.22038/IJBMS.2018.27721.6879

**Published:** 2018-07

**Authors:** Mohsen Abedi, Seyed Alireza Mesbah-Namin, Ali Noori-Zadeh, Taki Tiraihi, Taher Taheri

**Affiliations:** 1 Department of Clinical Biochemistry, Faculty of Medical Sciences, Tarbiat Modares University, Tehran, Iran; 2 Department of Clinical Biochemistry, Faculty of Paramedicine, Ilam University of Medical Sciences, Ilam, Iran; 3 Department of Anatomical Sciences, Faculty of Medical Sciences, Tarbiat Modares University, Tehran, Iran; 4 Shefa Neuroscience Research Center, Khatam-Alanbia Hospital, Tehran, Iran

**Keywords:** Expression, Ex-vivo, Gene delivery, Human SOD1, Rat BMSCs, Vector

## Abstract

**Objective(s):**

Human superoxide dismutase 1 (SOD1) is the cytosolic form of this enzyme it detoxifies superoxide anions and attenuates their toxicities and concomitant detrimental effects on the cells. It is believed that the amount of these enzymes present in the oxidative stress-induced diseases is crucial for preventing disease progression. Transfection of rat bone marrow stromal cells (BMSCs) by a constructed vector carrying the human wild-type *SOD1* gene, a non-viral gene transfer method, was the main aim of this study.

**Materials and Methods:**

For this purpose, the rat BMSCs were transfected with the vector using Turbofect reagent and then stabilized. Western-blot and real-time PCR were also used for evaluation of SOD1 expression.

**Results:**

Data analysis from RT-PCR and Western-blot techniques revealed that the stable transfected cells could secrete human wild-type SOD1 in the supernatant. Also, the total activity of SOD1 was about 0.5±0.09 U/ml and 0.005±0.002 U/ml in the supernatants of the transfected and not-transfected of rat BMSCs, respectively.

**Conclusion:**

This study showed that expansion of the stable transfected rat BMSCs by a constructed vector carrying the human wild-type *SOD1* gene is capable of secreting the active SOD1 enzyme under *ex-vivo *conditions. The recommendation of this study is that the same experiment would be applicable for expression of the other form of this enzyme, SOD3, as well. More valuable information could probably be provided about the variety of the diseases caused by superoxide anions toxicities by intervention and application of the non-viral method for expressions of SOD1 and SOD3 enzymes.

## Introduction

It is believed that aging is the consequence of molecular and cellular damage accumulations, leading to the functional decline of all cells with increased risk of disease and ultimately causing death (1). The free radical theory of aging indicates that the highly reactive chemical species derived from oxygen metabolism cause oxidative damage to various cellular molecules (especially initiate lipid peroxidation) leading to cellular aging (2). It is thought that 2% of the oxygen uptake by the cells is converted into reactive oxygen species (ROS) in the resting state (3). One aspect of ROS production is the intracellular activity of free radicals. Such free radicals can be involved in the subsequent biochemical reactions, producing additional molecules such as reactive nitrogen species (RNS), causing further damage to the cellular components (2, 4). Therefore, it is believed that ROS-induced oxidative damage is a key contributor to the reduced longevity of the cells and thus the organisms (5, 6). Overproduction of ROS and their derivatives occurs in the neurodegenerative diseases such as amyotrophic lateral sclerosis (ALS), multiple sclerosis, Parkinson’s disease (PD), Alzheimer’s disease (AD), and non-neurodegenerative diseases as well (7). It is also well accepted that superoxide anions play a crucial role in the etiology of these diseases (8-11). In fact, the dysfunctional aggregation in the non-native conformation of the proteins due to ROS production in these diseases leads to endoplasmic reticulum stress as well as mitochondrial dysfunction, thereby causing excessive production of ROS and further oxidative stress induction (12). The cell’s ability to deal with excessive ROS and RNS radicals requires the activation of pro-survival pathways as well as the production of molecules endowed with anti-oxidant and anti-apoptotic activities. These anti-oxidant defense systems include the superoxide dismutase, peroxidase enzymes, and glutathione as well. Therefore, in the healthy state, the cell becomes well equipped to cope with the normal production of reactive species (2, 4, 13). Indeed, continuous low concentrations of ROS induce expression of antioxidant enzymes and related defense mechanisms. However, the pathological actions of intracellular ROS occur when their concentrations are an order of magnitude higher than those physiological levels, causing various intracellular and subcellular component (e.g., lipids, proteins, and DNA) damages. Consequently, the amount of molecular damage increases with the advance of aging. In order to attenuate their concomitant detrimental effects, detoxifying of superoxide anions is carried out by the superoxide dismutase enzymes. These enzymes are a family of metalloenzymes that catalyze the dismutation of the superoxide anion to O_2_ and H_2_O_2_ and thus, they play a pivotal role in the cellular and extracellular antioxidant defense mechanisms (14, 15). In humans, three forms of superoxide dismutase (SOD) are present. SOD1 is predominantly localized in the cytoplasm and, to a lesser extent, in the nucleus; SOD2 is located in the mitochondria, and SOD3 is an extracellular isoform of this enzyme (14, 15). SOD2 has manganese in its reactive center, whereas SOD1 and SOD3 contain copper and zinc. SOD1 is a dimer of identical subunits, each about 16 KDa in size (14, 15). Regarding the important roles of superoxide dismutase enzymes as the anti-cytotoxic and anti-genotoxic factors in the body, SOD1 was selected in this study, due to its lower molecular weight, thereby making it more suitable for the subcloning process in comparison with other isoforms. Hence, we tried to construct a vector containing the gene encoding the wild-type of the human *SOD1* gene and analyzed its overexpression, secretion and the activity of the enzyme in the stably transfected rat BMSCs under *ex vivo *conditions. Particularly, in this field, it is of high interest to secrete SOD1 in the extracellular space, and it would be an alternative option to combat the extracellular ROS-mediated diseases.

## Materials and Methods

***Subcloning***

The carrier vector of the human wild-type *SOD1* gene, pINCY-h*SOD1, *GenBank accession number NM_000454, purchased from Open Biosystems company (clone Id: LIFESEQ2080042) and the human *SOD1 *gene on this vector was amplified using the PCR technique. The primers were designed so as to incorporate a *Hind *III and an *Xho *I restriction site in the forward and reverse primers, respectively, in which the restriction sites have been underlined and shown in Table 1. The pSecTag2/HygroB vector was digested with *Hind *III and *Xho *I restriction enzymes, then electrophoresed in a 1% agarose gel and then the fragment was purified using the MinElute Gel Extraction Kit (Qiagen, Germany). Human *SOD1 *gene PCR product was also digested with the same enzymes and purified with the Enzymatic Reaction Clean up Kit (Qiagen, Germany). The ligation reaction was performed using T4 DNA ligase (Fermentas, Lithuania) and then transformed into the *Escherichia coli* DH5α competent cells and the recombinant clones were screened on the agar plates supplemented with ampicillin (100 µg/ml). Positive colonies were confirmed via colony PCR and the recombinant pSecTag2/HygroB-human wild-type *SOD1* plasmid was extracted using the PureLink HiPure Plasmid Purification kit (Invitrogen, USA) and the nucleotide sequence of the human *SOD1* gene on the constructed vector was subjected to sequencing. All data in this research were analyzed using the Basic Local Alignment Search Tool or BLAST at http://www.ncbi.nlm.nih.gov. 

***Isolation and culture of rat BMSCs***

All surgical interventions and animal care procedures were approved by the Animal Ethics Committee in accordance with the guidelines for the care and use of laboratory animals prepared by Tarbiat Modares University, Tehran, Iran. Male inbred Wistar rats (220–250 g) were housed with free access to water and standard rat chow at controlled temperature (23°C) with daily exposure to a 12:12 hr light: dark cycle. After anesthetizing with ketamine and xylazine, whole bone marrows of the rats were extruded from the femur and tibia bones using an 18 G needle. This extracted bone marrow was centrifuged at 50 g for three min and the supernatant was collected and centrifuged at 300 g for five min. Then, the cell pellet was resuspended in α-MEM containing 10% fetal bovine serum (FBS, Gibco, USA), 2 mM L-glutamine (Gibco, USA), 100 U/ml of penicillin (Gibco, USA) and 100 µg/ml of streptomycin (Gibco, USA), then the cells were seeded in 6-well plates. After 48 hr, the non-adherent cells were removed by replacing the medium. Once the monolayer culture reached a conﬂuency of about 90%, cells were trypsinized with 0.05% Trypsin-EDTA (Gibco, USA) and plated again. 

***Immunocytochemistry***

 The rat BMSCs of the third passage were seeded in the 6-well plates (nearly 7000 cell/cm²). The cells were examined for purity by fibronectin and CD45 markers. They were washed three times, 5 min each with phosphate buffer saline (PBS, Gibco, USA), incubated in the fixative solution (4% paraformaldehyde in PBS) for 15 min and then washed with PBS again. They were treated with 0.3% Triton X-100 for 1 hr. For blocking the non-specific antibody binding sites at room temperature (RT), horse serum in PBS (10%) was used for 30 min. It was followed by incubation with the specific primary antibody (Chemicon, USA) in a 1:100 dilution for 1 hr and then washed twice, 5 min each with PBS. Subsequently, the incubation with fluorescein isothiocyanate (FITC) conjugated secondary antibody (Chemicon, USA) in 1:100 dilution was performed at RT for 1 hr. Then, the samples were washed twice, 5 min each with PBS. The nuclei of the cells were counterstained by ethidium bromide (Sigma, UK). The cells were digitally photographed using a fluorescence inverted microscope (Olympus, USA). The number of the immunoreactive cells was divided by the total cell number in order to estimate the percentage of immunoreactive cells. 

***Kill curve analysis***

 In order to produce stable cells expressing human SOD1 permanently, determination of the optimum antibiotic concentration that kills not-transfected rat BMSCs was necessary (there was the hygromycin B resistance gene on the pSecTag2/hygroB for stable transfection purposes in mammalian cells). Therefore, the rat BMSCs of the third passage were cultured on the 6-well plates and 0, 50, 100, 150, 200, and 400 concentrations of hygromycin B were added to each of the culture media. The media were exchanged with fresh media containing hygromycin B at aforementioned concentrations every 3 days after washing with PBS. 

***Cell transfection***

4×10^5^ rat BMSCs cells at the third passage were seeded in the 6-well plate 24 hr before the beginning of the transfection. The cells (95% confluent) were transfected with 4 μg of DNA/7 μl Turbofect Reagent (Fermentas, Lithuania) according to the manufacturer´s instructions, in which, a special medium known as Opti-MEM was used in the transfection steps to attenuate the adverse effects of serum on the transfection steps. In the Opti-MEM cell culture medium, the cells can survive without fetal bovine serum. However, we removed the serum from the medium gradually to adopt the cells to the new conditions. That is, after 4 hr of transfection, the medium was removed, and the fresh medium was added to the cells. Subsequent optimizations were performed for further increasing transfection efficiency. After 48 hr of transfection, hygromycin B was added to each well at the final concentration of 100 μg/ml for 10 days and during this time, the media were replaced with fresh medium containing fresh hygromycin B. 

***RT-PCR reaction***

Total RNA was isolated from stable transfected rat BMSCs and also from not-transfected cells (third passage) using an RNA extraction kit (Invitrogen, USA) according to the manufacturer’s instructions. Subsequently, DNase I (Invitrogen, USA) treatments were followed. The purified total RNA was subjected to cDNA synthesis using RevertAid first strand cDNA synthesis kit (Fermentas, Lithuania) in the transfected as well as not-transfected cells. The PCR amplifications were performed using the 2X PCR master mix (Fermentas, Lithuania) with 0.5 μl of reverse transcription product for each sample of human *SOD1* with rat glyceraldehyde-3-phosphate dehydrogenase (*GAPDH*, housekeeping gene) and *SOD1* primers which have been listed in Table 2. 

***Western blot***

 The supernatant of the transfected cells was collected on ice, and protease inhibitor cocktail (Fermentas, Lithuania) was added immediately to the samples. Then, protein concentration was assessed by Bradford assay. 30 µl of the cell supernatant was diluted in the 4X sample buffer, boiled at 95 ^°^C for 10 min and then subjected to SDS-PAGE with the gradient gel running from 4 to 12% acrylamide gel. After electrophoresis, the gel was transferred onto the nitrocellulose membrane (Millipore, USA) using the semi-dry method (BioRad, USA) and then, the membrane was blocked by 10% BSA in PBS for 2 hr at RT. Then, it was incubated overnight with mouse anti-human SOD1 monoclonal primary antibody (sc-101523, Santa Cruz, USA) diluted with 1% BSA+PBS (1:100). After three times washing, 15 min each with TBST, the membrane was incubated with the HRP-conjugated goat anti-mouse secondary antibody (sc-2060, Santa Cruz, USA). For detection of the protein band in the membrane, DAB (Diaminobenzidine) solution and hydrogen peroxide were used as the substrate for the conjugated peroxidase enzyme. The human SOD1 band appeared after 5–15 min of substrate additions to the membrane.

***SOD1 activity assay***

 In order to determine the SOD activity in the supernatants of the transfected and not-transfected rat BMSCs, we assessed the SOD activity of the aforementioned cells utilizing the SOD activity assay kit (SOD Assay Kit-WST, Japan), which allows SOD assay using Dojindo’s highly water-soluble tetrazolium salt, WST-1 (2-(4-Iodophenyl) -3- (4-nitrophenyl) -5 - (2,4-disulfophenyl) -2H-tetrazolium, monosodium salt), which produces a formazan dye upon reduction with the superoxide anion. The rate of the reduction of WST-1 with O2•^-^is linearly related to the xanthine oxidase (XO) enzyme activity, and this reduction is inhibited by SOD1. In this assay, the rate of the O_2_ reduction is linearly related to the XO activity, and inhibited by SOD1. Therefore, IC_50_ (50% inhibition activity of SOD) can be determined by a colorimetric method at 450 nm at RT. The reactions were followed according to the manufacturer’s instructions and each sample was prepared in triplicate and using a microplate reader the absorbance at 450 nm after 20 min was recorded. The SOD1 activity (inhibition rate %) was calculated using the mentioned equation in the manufacturer’s instructions. 

**Figure 1:**
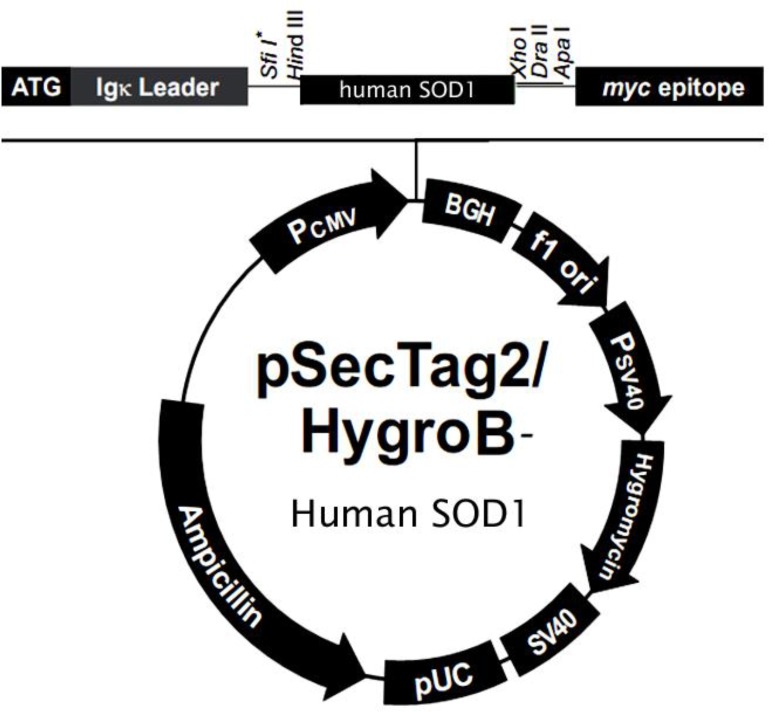
The human wild-type SOD1 mammalian expression vector construction. Human wild-type SOD1 was subcloned into the expression vector after murine Ig-Kappa chain V-J2-C signal peptide in the pSecTag2/HygroB between the Hind III and Xho I restriction sites to construct the pSecTag2/HygroB-human wild-type SOD1 expression vector

**Figure 2:**
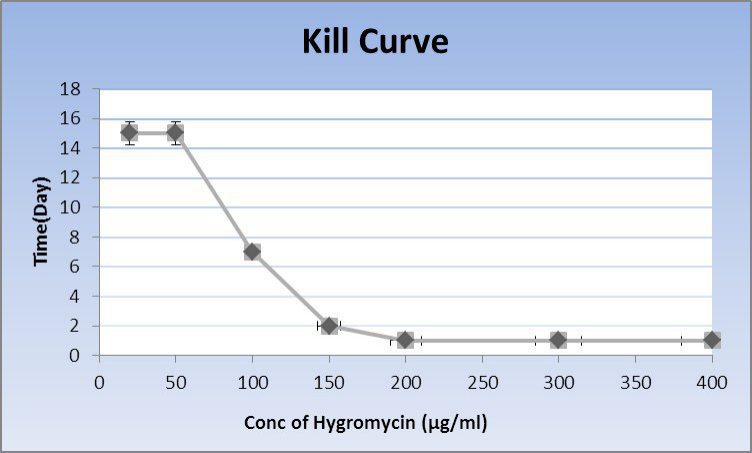
Kill curve. The kill curve for the determination of the optimal concentration of the antibiotic that killed the not-transfected rat BMSCs. The optimal concentration of hygromycin B that could kill the not-transfected cells after 7 days of culture was assessed using kill curve analysis and the results showed that 100 μg/ml was optimum

**Figure 3:**
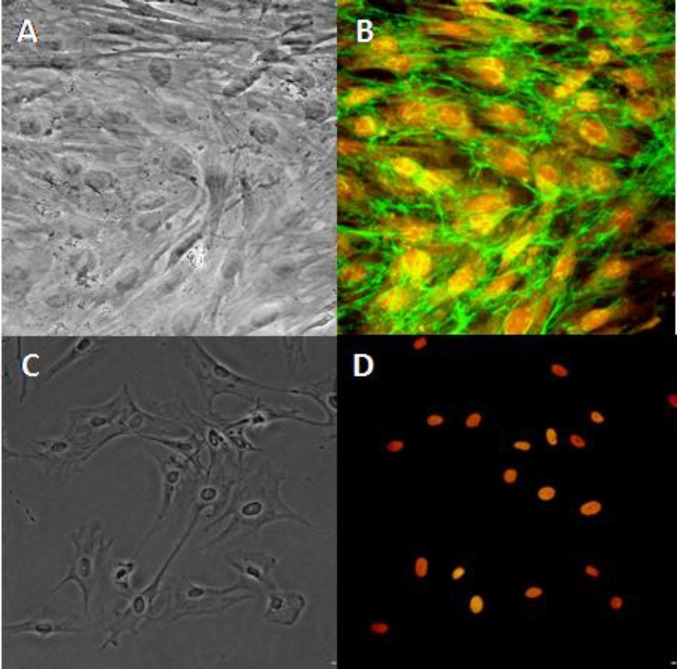
The immunocytochemistry pattern of the isolated rat BMSCs. The left panel represents phase contrast of the cultured rat BMSCs (A×200 and C×100) and the right panel shows fibronectin (B×200) and CD45 (D×100) expressions at the third passage of the cells. The expression of each protein was detected by a specific primary antibody followed by a suitable FITC-conjugated secondary antibody. The immunoreactivity percentage (±SEM) of CD45 and fibronectin were 1 ±0.2 and 99.9±0.1, respectively. The cell nuclei were counterstained with ethidium bromide

**Figure 4:**
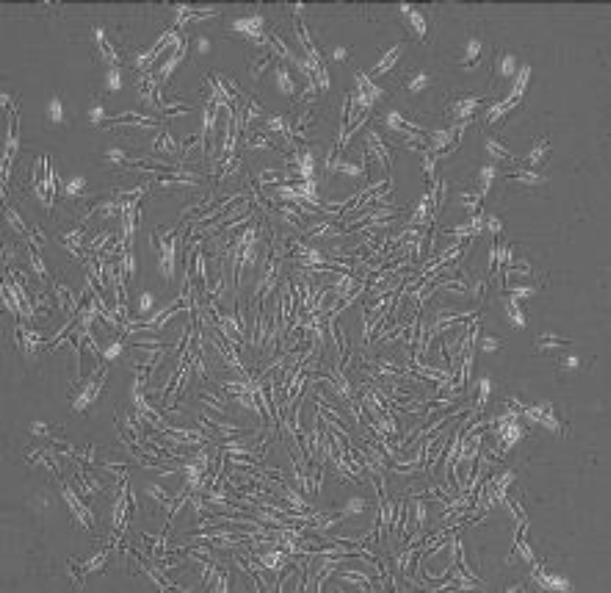
The hygromycin B-resistant transfected rat BMSCs colony (×100). The rat BMSCs transfected with the pSecTag2/HygroB-human wild-type *SOD1* vector were cultured in α-MEM complete medium containing 100 µg/ml hygromycin B for 10 days to form the resistant cells that can stably express the human wild-type *SOD1* gene

**Figure 5:**
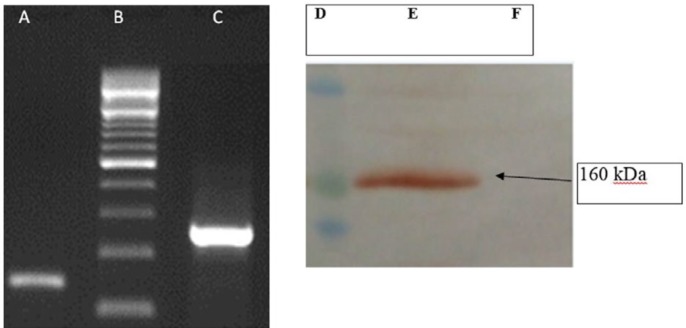
The RT-PCR and Western-blot results. Left panel represents agarose gel electrophoresis for the gene expression of the stable transfected rat BMSCs by RT-PCR. Lane A: human wild-type SOD1; Lane B: DNA ladder marker, Lane C: GAPDH. Right panel represents the Western-blot results for the human wild-type SOD1 protein expression in the supernatant of the stable transfected rat BMSCs. The band with about 16 kDa was detected on the blotting membrane for this protein. Lane D: protein ladder marker, Lane E: transfected BMSCs; Lane F: not-transfected BMSCs

**Figure 6:**
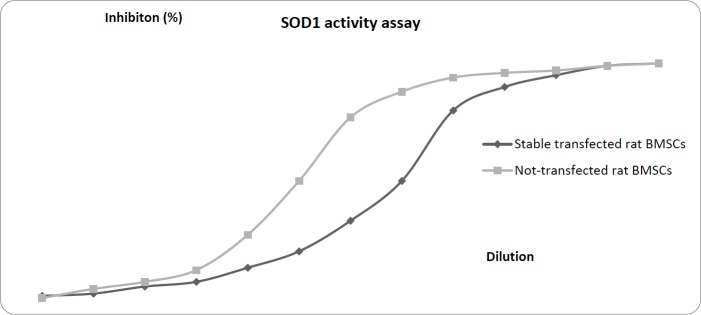
The SOD1 activity assay in the supernatant of the not-transfected and stable transfected rat BMSCs. The results showed that total SOD1 activity was about 0.5±0.09 U/ml and 0.005±0.002 U/ml in the supernatant of the transfected and not-transfected rat BMSCs, respectively

**Table 1:** The primers that were used for PCR amplification of the human wild-type SOD1 gene from the carrier vector (underlined sequences are the enzyme restriction sites)

Primer name	Sequence	Product size (bp)	Annealing temperature (°C)
Human SOD1	5' GGGGAAGCTTGTTATGGCGACGAA 3' 5' CCGCTCGAGTGTTTATTGGGCGAT 3'	490	64.4

**Table 2:** The primers that were used for the RT-PCR reactions

Primer name	Sequences	Product size (bp)	Annealing temperature (°C)
Human SOD1	5´ GCATCATCAATTTCGAGCAGAAG 3´	310	66
	5´ CTTTTTCATGGACCACCAGTGTG 3´		
Rat GAPDH	5´ CAAGGTCATCCATGACAACTTTG 3´	496	58
	5´ GTCCACCACCCTGTTGCTGTAG 3´		

## Results

***Subcloning of the human SOD1 in the expression vector***

The gene encoding human *SOD1* from the primary pINCY-h*SOD1 *vector was successfully subcloned into the pSecTag2/HygroB expression vector. The PCR product was inserted between *Hind III* and *XhoI* restriction sites of pSecTag2/hygroB creating a constructed vector with a murine Ig-Kappa chain V-J2-C leader sequence at the N-terminal of the human *SOD1* gene (Figure 1). Sequencing data analysis of the inserted human *SOD1* in the pSecTag2/HygroB-human wild-type *SOD1* vector confirmed the sequence validity of the inserted gene as published by GenBank bioinformatics database. 

***Kill curve***

 The optimum concentration of hygromycin B, which could kill the not-transfected cells after 7 days of culture was about 100 μg/ml (Figure 2).

***Immunocytochemistry of rat BMSCs***

The immunocytochemistry pattern showed that the purity of the rat BMSCs was high. The immunoreactivity of CD45 and fibronectin markers was 1 ± 0.2 % and 99.9 ± 0.1%, respectively (Figure 3). 

***Stable transfection***

 By addition of hygromycin B (100 μg/ml) into the culture medium, the individual antibiotic-resistant rat bone marrow stromal cell colony appeared and these cells were then subjected to further gene expression analysis (Figure 4). 

***RT-PCR results***

 The results of RT-PCR showed that human *SOD1* gene is expressed in the stably transfected rat bone marrow stromal cells (Figure 5).

***Western-blot results***

Western-blot analysis of the cell supernatant with a primary human-specific monoclonal antibody that only recognizes human SOD1 (Figure 5) showed the successful stable transfection of rat bone marrow stromal cells and secretion of the human SOD1 from the cells. 

***Total SOD1 activity assay results***

According to the instructions provided by the manufacturer of the kit, SOD1 activity (inhibition rate %) was calculated using the following equation: *SOD *activity (inhibition rate %) = [(A_blank 1_- A_blank 3_) – (A_sample_ - A_blank 2_)] / [A_blank 1_-A_blank 3_] x 100. The results showed that total SOD1 activity was nearly 0.5±0.09 U/ml and 0.005±0.002 U/ml in the supernatant of the transfected and not-transfected rat BMSCs, respectively (Figure 6). 

## Discussion

In this research, we aimed to find a way for enabling stem cells to secrete this important enzyme by constructing a vector capable of secreting human SOD1 into the extracellular matrix. We stably transfected rat BMSCs with the constructed pSecTag2/HygroB-human wild-type *SOD1* vector using a non-viral method and also evaluated the functionality of the secreted human wild-type SOD1 to the supernatant of the cells using the SOD1 activity assay and other molecular techniques. This is why, in this research, the transfected BMSCs have the potential therapeutic applications for detoxifying extracellular generated superoxide anions and thus promoting cell membrane integrity, functionality, and finally preventing cell death in a variety of ROS-mediated diseases. The presence of sufficient amounts of this enzyme in the cells and also tissues is vital and typically keeps the concentration of superoxide at the minimum concentration. Furthermore, for the enzyme functionality evaluation, we assessed total SOD1 activity, and in this regard, data showed that its total activity was nearly 0.5 U/ml and 0.005 U/ml in the supernatant of the transfected and not-transfected rat BMSCs, respectively. Indeed, it clearly revealed that the enzyme is functionally active. Moreover, as BMSCs can secrete a variety of neurotrophic factors *per ce* and indeed, several lines of evidence show that they can secrete glial cell line-derived neurotrophic factor (16, 17), nerve growth factor (17), brain-derived neurotrophic factor (18), neurotrophin 3, as well as neurotrophin 4 (19). It seems that transfected BMSCs capable of secreting SOD1 are a suitable source of combined therapy for the central nervous system disorders due to the neuronal support provided by neurotrophin secretion from such cells as well as detoxifying and neutralizing superoxide anions by SOD1 secretion. These properties make such cells a suitable candidate for the treatment of neurological diseases. Thus, in particular, the *SOD1* gene appears to be important in the prevention of the neurodegenerative disorders such as AD (20), PD (21), HD (22), and ischemia as well (23, 24); since studies showed that inhibition or down-regulation of SOD activity can result in apoptosis of the neuronal cells (25-27). Also, from another point of view, further applications of SOD1 secreting stem cells are their utility in the cardiovascular diseases, including ischemic heart disease, cerebrovascular disease, atherosclerosis, and other related problems, such as myocardial infarction. Reduction of the cardiovascular disease has been documented in several epidemiological studies in individuals supplemented with antioxidants (7, 28). To oxidized low-density lipoproteins (Ox-LDLs) have been attributed numerous pro- and anti-atherogenic properties. The Ox-LDLs migrate across the endothelial membrane into the arterial walls and these oxidized components attract macrophages, which absorb and deposit cholesterol within the cells to form foam cells. From this aspect of view, using soluble SOD1 in the body fluids can moderate such reactions and LDL oxidation, which renders the body resistant to cardiovascular problems. The important aspect of the present study was using human wild-type of* SOD1* gene, in contrast to other studies that used the mutant form of the SOD1 enzyme, which could trigger the characteristics of ALS in the corresponding cells in order to investigate the adverse effects of the mutant form of this enzyme (29-31). However, a similar study demonstrated the efficacy of SOD1 in the treatment of cataracts, as the overexpression of *SOD1* in the whole lens prevents H_2_O_2_-induced oxidative damage to the lens (32). Meanwhile, in recent similar research work on *SOD1* and *SOD3* gene transferring indicated that adenoviral-mediated gene transfer of *SOD3*, but not *SOD1*, could improve endothelial-dependent relaxation and protect the aorta from xanthine/ xanthine oxidase-mediated endothelial dysfunction. It also emphasized that the location and enzymatic source of superoxide anion production is an important insight in vascular disease (33). It is notable that we used the non-viral method for expansion of the stable transfected BMSCs enabled to secrete the human wild-type SOD1 in the supernatant, which had considerable specific activity and on the other hand, this method is safer for the BMSCs. 

## Conclusion

This study showed that expansion of the stable transfected rat BMSCs by a constructed vector carrying the human wild-type *SOD1* gene is capable to secret the active SOD1 enzyme compared with non-transfected cells under *ex-vivo* conditions. Also, this experiment would be applicable for expression of the extracellular forms of this enzyme, such as SOD3, as well. In this regard, valuable information could probably be provided about the variety of the diseases caused by superoxide anions toxicities by intervention and application of the non-viral method for expressions of SOD1 and SOD3 enzymes. 

## Conflicts of interest

The authors declare that they have no conﬂicts of interest.
